# Ultrasound-Guided Supraclavicular Nerves Block for Acute Pain Management in Clavicular Fractures—A Pragmatic Randomized Trial

**DOI:** 10.3390/jcm14228249

**Published:** 2025-11-20

**Authors:** Eckehart Schöll, Mark Ulrich Gerbershagen, Werner Vach, Maria Rösli, Rainer Jürgen Litz

**Affiliations:** 1 Department of Emergency Medicine, Bethesda Spital, 4052 Basel, Switzerland; 2 Faculty of Medicine, University of Witten Herdecke, 58455 Herdecke, Germany; 3 Department of Anaesthesiology, Hospital Cologne Holweide, 51067 Cologne, Germany; 4 Basel Academy for Quality and Research in Medicine, 4051 Basel, Switzerland; werner.vach@basel-academy.ch; 5 Spital Emmental, 3400 Burgdorf, Switzerland; maria.roesli@spital-emmental.ch; 6 Independent Researcher, 86199 Augsburg, Germany; r.j.litz@usra.de

**Keywords:** regional anesthesia, ultrasound-guided block, supraclavicular nerve, clavicle fracture

## Abstract

**Background/Objectives:** This pragmatic randomized controlled trial evaluated the efficacy of ultrasound-guided supraclavicular nerve (SCLN) block compared to standard pain management in patients with acute displaced clavicle fractures (CFs) in an emergency department (ED) setting. Secondary outcomes included time to first request for analgesics, opioid consumption, and patient satisfaction. **Methods:** Forty-one patients with acute displaced CFs were randomized to receive either an SCLN block (*n* = 19) or routine pain management (*n* = 22). Pain intensity was recorded at admission and at 1, 2, 4, 6, 12, and 24 h. Patient satisfaction was assessed after 24 h. Analgesic use, adverse reactions, and adverse events were documented for 24 h. **Results:** Pain intensity, measured by the numeric rating scale (NRS), was significantly lower in the SCLN group at all time points within the first 12 h (*p* < 0.001). After one hour, 68% of patients in the SCLN group reported an NRS of 0–2, compared to 19% in the control group. The time to first request for analgesics was markedly longer in the SCLN group (9.1 h vs. 0.7 h). In two patients, SCLN visualization was insufficient, and a cervical plexus block was performed instead. Four patients in the SCLN block group reported adverse reactions. Patient satisfaction after 24 h was significantly higher in the SCLN group (*p* < 0.001), with 85% indicating they would choose the block again. **Conclusions:** Ultrasound-guided selective SCLN block appears to be an effective and well-tolerated method for acute analgesia in patients with displaced CFs, with the most pronounced benefit observed during the first 12 h. Patient acceptance of the procedure was high.

## 1. Introduction

Clavicular fractures (CFs) belong to the ten most common fractures in adults and occur particularly often in men during cycling or recreational activities [1,2]. Undisplaced CFs are usually treated conservatively, whereas displaced fractures frequently require surgical fixation to achieve optimal functional outcomes [3,4]. Regardless of fracture morphology, the initial pain can range from moderate to severe and often poses a challenge during early medical treatment [5]. Consequently, emergency physicians, orthopedic and trauma surgeons, and anesthesiologists are frequently required to provide effective analgesia for patients presenting with acute CFs. Multimodal systemic analgesic regimens are typically used, but their efficacy may vary, and opioids [6,7], in particular, can cause unwanted side effects such as drowsiness, nausea, or vomiting. In this context, selective peripheral nerve blocks (PNBs) may offer a straightforward and potentially opioid-sparing alternative for acute pain management in the emergency department (ED).

PNBs have been widely described for clavicle surgery, either as adjuncts to general anesthesia or as sole anesthetic techniques. Cadaveric studies have demonstrated that the clavicle receives sensory innervation from several nerves, including the subclavian nerve, the lateral pectoral nerve, and the supraclavicular nerves, arising from both the brachial plexus and the cervical plexus [8]. Effective anesthesia for clavicle surgery, therefore, often requires a combination of blocks targeting both plexuses [9,10,11]. The combination of an interscalene brachial plexus block (ISB) and a cervical plexus block (CPB) is recommended as the regional technique of choice for surgical anesthesia of the clavicle [12].

For acute pain management in the ED, however, the aims and risk–benefit considerations differ from those during anesthesia for surgery. An ISB carries a relevant risk of phrenic nerve (PN) involvement and may cause transient respiratory impairment. Investigations of PN-sparing PNB techniques after shoulder surgery highlight the importance of minimizing unintended diaphragmatic dysfunction [13]. Although an accurately performed intermediate CPB should theoretically avoid PN involvement, it still anesthetizes additional cervical plexus branches such as the great auricular nerve (GAN), transverse cervical nerve (TCN), and lesser occipital nerve (LON), which may be unnecessary for analgesia and may influence patient comfort.

Since the supraclavicular nerves (SCLNs) are the main sensory providers to the clavicle [8,9,14] and can be reliably visualized using high-resolution ultrasound imaging (HRUI), we hypothesized that an ultrasound-guided selective SCLN block could improve initial pain management in patients with acute CFs. We therefore conducted this randomized controlled trial to evaluate the analgesic effect of an SCLN block compared to standard ED management without regional anesthesia (RA).

## 2. Materials and Methods

### 2.1. Study Design and Ethics

This single-center, parallel, randomized controlled clinical trial compared an ultrasound-guided SCLN block with conventional analgesic management for acute CFs in an ED. Adult patients (≥18 years) presenting with isolated CFs scheduled for surgical treatment were eligible. Exclusion criteria included contraindications to RA (e.g., local infection, allergy to local anesthetics), multiple trauma, inability to cooperate, chronic analgesic use, or neuromuscular disorders.

The study was approved by the regional Ethics Committee (BASEC2020-03054) and registered at ClinicalTrials.gov (NCT04685291). All participants provided written informed consent. The trial followed the Declaration of Helsinki and the CONSORT guidelines [15].

### 2.2. Randomization

Patients were randomized 1:1 to the block or control group using a computer-generated allocation sequence prepared by an independent statistician. Allocation numbers were sealed in opaque envelopes and opened by a study nurse not involved in patient assessment. Blinding was not feasible due to the obvious sensory effects of the block.

### 2.3. Sample Size

Based on prior casuistic experience indicating a distinct analgesic effect, we assumed an effect size of Cohen’s d = 0.8. Under this assumption, 21 patients per group were required to achieve 80% power using a one-sided *t*-test at a significance level of 5%.

### 2.4. Ultrasound-Guided Supraclavicular Nerves Block

All blocks were performed in the ED by providers trained in ultrasound-guided PNBs. Training consisted of theoretical instruction in relevant sonoanatomy, hands-on ultrasound practice on healthy volunteers, and supervised performance of ultrasound-guided PNB in at least three study patients.

Patients were positioned supine with the head slightly rotated away from the affected side. A high-frequency linear transducer was placed in the posterior cervical triangle to identify the sternocleidomastoid muscle and key fascial layers. The SCLN cluster was visualized as it emerged from the cervical plexus. Detailed sonoanatomical descriptions and procedural illustrations are provided in the Supplementary Material (Supplemental Figures S1–S3).

Local anesthetic (LA) was injected in-plane from lateral to medial, strictly above the prevertebral fascia. The intended volume was 3 mL of bupivacaine 0.5% mixed with 75 µg clonidine [16]. Blocks were considered successful if pain decreased to NRS ≤ 3 within five minutes. If SCLN visualization was insufficient, an intermediate cervical plexus block was recommended.

### 2.5. Pain Medication Protocols

Before allocation, all patients received standard ED analgesia (ibuprofen 400 mg and metamizole 1000 mg, orally or intravenously). Patients with intolerance received paracetamol 1000 mg.

Control group patients continued this regimen (ibuprofen every 8 h; metamizole every 6 h; acemetacin 60 mg instead of ibuprofen for patients >70 years). Oxycodone was available as rescue medication for severe pain.

In the block group, no routine basic analgesia was administered after the block. On request, patients received ibuprofen or acemetacin (first-line), metamizole (second-line), and oxycodone for severe pain.

### 2.6. Outcome Measures and Data Collection

Primary outcomes were (1) pain intensity and (2) patient satisfaction. Pain was rated using an 11-point numeric rating scale (NRS) at admission and at 1, 2, 4, 6, 12, and 24 h. Satisfaction was assessed after 24 h using a structured questionnaire (Supplemental Table S1).

Secondary outcomes included time to first analgesic request, 24 h analgesic consumption, adverse reactions and adverse events, frequency of surgery within 24 h, and discharge within 24 h.

Baseline characteristics (age, sex, ASA status, BMI, accident mechanism, dominant side, and fracture classification according to Allman [17]) were extracted from patient records. The Allman classification was chosen because it is anatomically most suitable for the context of regional anesthesia and aligns well with comparative analyses of clavicle fracture classification systems [18].

### 2.7. Statistical Analysis

Pain intensity was summarized at each time point using mean values and visualized with stacked bar charts. Group differences were tested using Wilcoxon rank-sum tests. Pain-free duration within 12 h was estimated by interpolation between time points and depicted using cumulative distribution functions. Time to first analgesic request was analyzed using the Kaplan–Meier estimator, with hospital discharge treated as a censoring event.

For each analgesic, frequency of use and cumulative 24 h dose were reported. Adverse events and adverse reactions were summarized descriptively.

## 3. Results

### 3.1. Recruitment, Randomization, and Patient Characteristics

**Figure 1:**
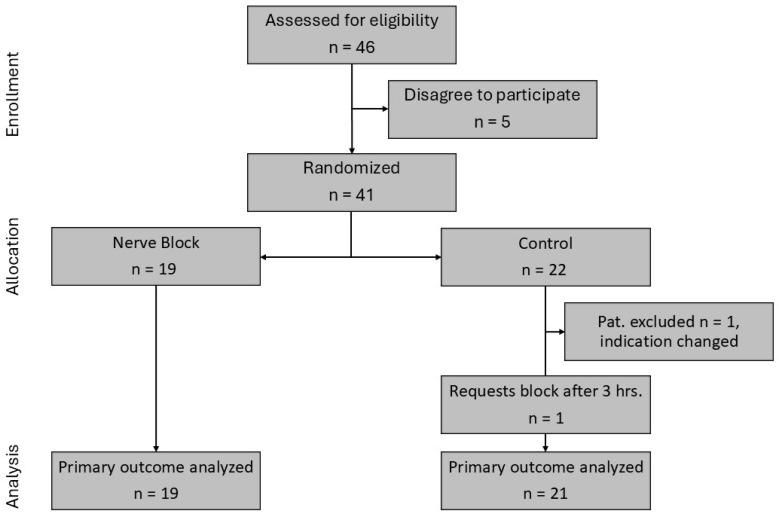
CONSORT study flowchart.

Patients were recruited between 15 April 2021 and 6 July 2023, when the intended sample size was nearly reached. According to Figure 1, 46 patients were eligible, and 41 patients were randomized. One patient was excluded from the analysis due to a change of indication after randomization. One patient in the control group requested a block after 3 h. This patient remained in the control group. 19 patients were available for analysis in the block group, and 21 patients were available for analysis in the control group.

Patients had an ASA physical status between 1 and 3, were aged between 18 and 71 years, and were predominantly male. There were no significant differences in patient demographics and clinical characteristics between the groups (Table 1).

### 3.2. Block Performance

Table 2 depicts the distribution of some characteristics of the block. In two patients (11%), the SCLN was not clearly identified, and an intermediate CPB was performed. In two patients, ropivacaine instead of bupivacaine was used as LA, and in one patient, clonidine was omitted. In the majority of patients, the actual dose of the LA was above the intended dose, with an average value of 3.8 mL. On average, the block was performed 26 min after consent and 52 min after admission.

### 3.3. Pain Relief

In all patients, a pain assessment was performed at all planned time points. The individual pain courses are depicted in Supplemental Figure S4 in the online Supplemental Material. In the block group, often an early distinct decline could be observed, whereas in the control group, many patients remained at their initial pain level for a longer period.

**Figure 2:**
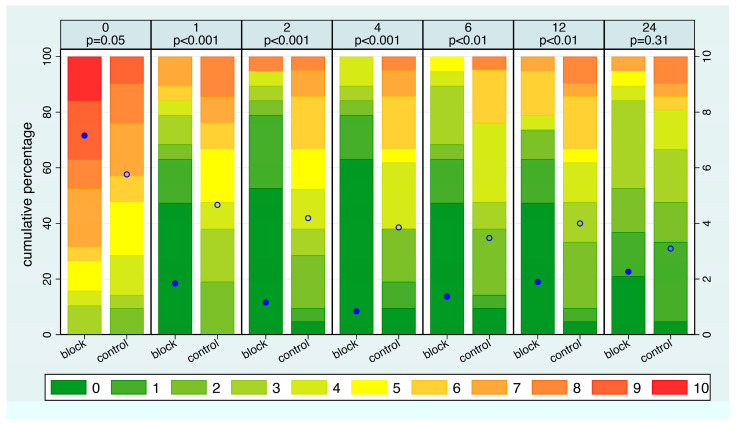
The distribution of pain intensity at each time point, stratified by intervention group. The eleven possible pain levels are represented by different colors, from green to red. The mean pain intensity at each time point is shown as a blue dot, which refers to the NRS on the right *y*-axis. *p*-values refer to a comparison of the two intervention groups. Filled dot: block group. Open dot: control group.

Considering the distribution of pain intensity at each time point (Figure 2), NRS ratings at admission were slightly higher in the SCLN block group compared to the control group (*p* = 0.05). In the SCLN block group, the average pain intensity significantly decreased within just one hour and continued to decline gradually until the fourth hour. In contrast, in the control group, the average pain intensity decreased very slowly over time. In the block group, the average pain intensity increased slowly after 4 h but remained lower than that of the control group. The difference in pain intensity was significant at all time points except after 24 h. With respect to the distribution of the pain intensity, after 1 h, 68% of patients in the SCLN group reported an NRS of 0–2, compared to 19% in the control group. Furthermore, at any time point between one hour and twelve hours, more than 40% of patients in the block group reported an NRS score of 0. In contrast, more than 40% of patients in the control group reported an NRS score of 5 or higher at 1, 2, and 4 h. Half of all patients in the block group had six hours free of pain, whereas this never happened in the control group. Additionally, nearly 80% of patients in the block group experienced a maximum pain level of 3 or lower for at least 8 h, compared to only one-third of patients in the control group (Supplemental Figure S5).

The median time from signing the consent until requiring pain medication was 0.7 h in the control group and 9.1 h in the block group. 54% of patients in the control group required pain medication within 1 h, whereas this was the case in only one patient in the block group.

### 3.4. Patient Experience

**Figure 3:**
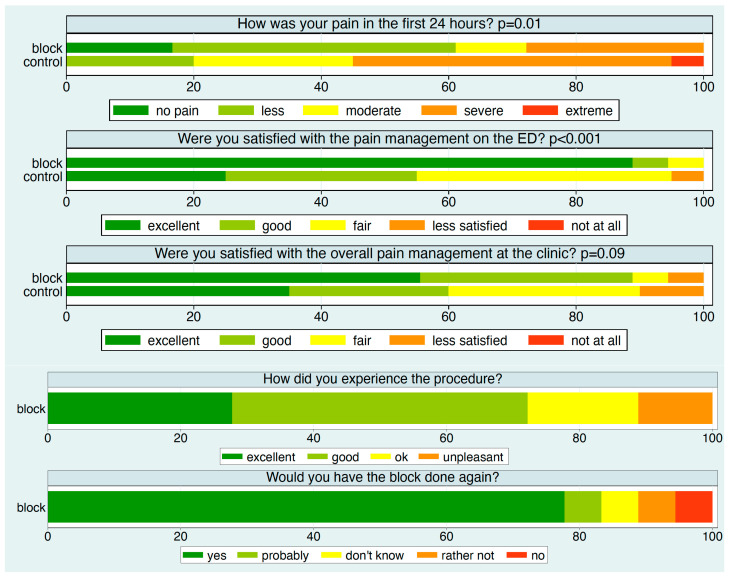
The distribution of the patient experience measures at each time point stratified by intervention group. *p*-values refer to a comparison of the two intervention groups.

Except for two patients—one in each group—all participants reported their experience after 24 h (Figure 3). Approximately 90% of patients in the block group rated their satisfaction with pain management in the ED as excellent, compared to about 25% in the control group. Specifically, over 60% of patients in the block group reported no or minimal pain, compared to 20% in the control group.

Satisfaction with overall pain management in the hospital was also higher in the block group. In this group, about 75% of patients rated the block procedure as excellent or good, and nearly 80% would choose the block again.

### 3.5. Pain Medication

Table 3 shows the frequency and dosage of all types of pain medication administered. A clear trend is evident towards less frequent use of pain medication and lower doses in the block group compared to the control group with the exception of oxycodone. Ketorolac and fentanyl had been given to some patients, deviating from the intended pain management, which reflects the need for additional management options in some patients. A post hoc check of the use of oxycodone indicated an occasional preference in order to support the sleep of patients.

### 3.6. Adverse Reactions and Adverse Events

In both groups, patients did not report any adverse events related to the pain medication. Four patients in the block group reported adverse reactions, indicating a block in the area of the GAN or TCN. Two of these patients had received an intermediate CPB, while the other two had received LA doses of 4 and 5 mL, respectively. In three patients, the sensation was classified as mild. The fourth patient felt bothered by temporary numbness in the ear on the affected side.

### 3.7. Impact on Patient Management

Surgery was performed within 24 h in 13 of the 19 patients (68%) in the block group, compared to 11 of the 21 patients (57%) in the control group. Patients were discharged home within 24 h more often in the control group (2/19 vs. 11/21).

## 4. Discussion

This is the first randomized controlled trial evaluating the efficacy of a selective SCLN block for pain relief during the initial treatment of acute CF. Our findings indicate that an ultrasound-guided selective block of the SCLN can rapidly reduce acute pain to a substantial degree without causing major side effects. The effect was most pronounced within the first 12 h and resulted in significantly fewer early requests for analgesics. Previous trials in RA have mainly focused on surgical anesthesia rather than on analgesia during the early, non-operative phase of treatment in the ED [12]. Yet, effective pain relief is crucial during this period, which includes diagnostics, potential reduction in dislocation, and immobilization. Initial pain intensity may range from moderate to severe and can occasionally be unresponsive to opioids [19].

For surgical anesthesia, blocking branches of both the brachial plexus (pectoral nerves, subclavius nerve) and the cervical plexus (supraclavicular nerves) is required due to the complex innervation of the clavicle [8,9]. Several RA techniques combining ISB, CPB, or fascia plane blocks have therefore been described. While generally effective, techniques involving an ISB may be associated with side effects or complications, particularly due to unintended PN involvement [20]. In contrast, intermediate CPB with higher LA volumes may provide adequate analgesia without blocking the PN [21]. In our study, we demonstrated that the topography of the SCLN allows for a selective block guided by ultrasound. The SCLN, which typically emerges from the C4 ventral ramus alongside the PN, can be visualized as it courses beneath the prevertebral fascia (PVF) and subsequently perforates it, whereas the PN remains underneath the PVF [14]. After piercing the PVF, the branching pattern of the SCLN (medial, intermediate, lateral) can be depicted, although its variability occasionally complicates identification. This may explain why the SCLN could not be clearly visualized in two patients with challenging ultrasound conditions.

Within the interfascial space, SCLN branches share the compartment with other sensory cervical plexus branches, such as the GAN, TCN, and LON, mainly deriving from C2 and C3. This anatomical relationship explains inadvertent blockade of these branches through uncontrolled LA spread, as observed in two patients in our study. Although usually clinically benign, PN involvement has recently been described even with intermediate CPB [22], particularly when higher LA volumes (e.g., 10 mL) are used. We attempted to minimize this risk—which is especially relevant in patients with pre-existing pulmonary disease [20]—by using a low LA volume and targeting the injectate strictly above the PVF. For clavicle surgery, LA volumes of up to 10 mL are commonly used [10,23], and even higher volumes are applied in carotid surgery [22,24], although such high doses appear unnecessary for acute CF analgesia. Very low LA volumes (<1 mL) have produced adequate peripheral nerve blocks in other settings [25]. Given the unpredictable spread within the interfascial space, we intended to inject 3 mL close to the SCLN. The actual injected dose was occasionally higher, which may explain the unintended blockade of GAN or TCN in two patients. Although not harmful, this affected comfort and generated some uncertainty.

The two patients in whom SCLN visualization was insufficient received an intermediate CPB but did not obtain effective pain control within two hours, with pain levels of 4 and 7. This underscores the unpredictability of LA spread within the fat- and connective-tissue–rich interfascial space. Whether higher LA volumes would have produced a reliable block in these cases remains speculative.

As expected, ultrasound-guided RA of the SCLN proved to be an effective method for reducing pain intensity in the initial treatment of acute CF and significantly enhanced patient satisfaction. Patients in the block group frequently reported maximum NRS values of 3 during the first 12 h, a level rarely achieved in the control group. The frequent administration of LA volumes exceeding the protocol specification may partly reflect the additional, but undocumented, skin infiltration prior to block placement. It may also indicate some initial skepticism among ED physicians regarding the efficacy of lower LA volumes. Likewise, the common decision to keep patients in the hospital after receiving the block may reflect cautious observation, which proved justified to some extent due to the gradual loss of analgesic efficacy over time. Whether strict adherence to a 3 mL dose could reduce adverse reactions while preserving analgesic effectiveness should be further explored. These questions can be addressed through future observational studies. In cases where the SCLN cannot be clearly visualized beneath the PVF, improved management strategies are needed, including determining how best to address decreasing block efficacy over time.

This study has several limitations. Reliable information on pre-admission analgesia was unavailable. Block administration did not occur at a standardized time point after admission, which may have influenced initial NRS scores. Our focus was on the clinical effect rather than detailed sensory mapping. Due to the proximity of the C5 and C6 nerve roots, LA spread into the brachial plexus territory cannot be fully excluded, even with low LA volumes, making it difficult to confirm that a purely selective SCLN block was achieved. Moreover, although we performed clinical assessments to detect PN involvement, neither ultrasound nor functional respiratory measurements were conducted. We did not measure block onset time, although previous observations and reports suggest it is typically less than 5 min [23]. Clonidine was used as an adjuvant, although its effect remains controversial, with some studies reporting prolonged block duration [26,27,28] and others not confirming a benefit [16,29,30]. Pain medication use was only controlled during the patients’ ED stay; therefore, the frequency of rescue medication could not be meaningfully compared between groups. Finally, patients receiving a block were seldom discharged early, exposing them more frequently to hospital-based management, including the use of oxycodone to support sleep.

Overall, when interpreting the results, the lack of blinding, the relatively small sample size, and potential sources of selection bias should be taken into account. Larger multicentre studies will be required to confirm the effectiveness and safety profile of this technique.

## 5. Conclusions

Ultrasound-guided blockade of the supraclavicular nerves appears to be an effective and well-tolerated option for reducing pain intensity and improving patient satisfaction in patients presenting with acute clavicular fractures in the emergency department. Patients should be appropriately informed about possible side effects, which may potentially be minimized through strict dose control and careful technique.

## Figures and Tables

**Table 1:** Patient characteristics and classification of fractures. Data are presented as mean (range), or numbers (percentage) if applicable.

	Block (*n* = 19)	Control (*n* = 21)	*p*-Value
Age (years)	40 (18–70)	43 (18–67)	0.56
Female/male	1 (5%)/18 (94.7%)	2 (9.5%)/19 (90.5%)	1.00
ASA	1.5 (1–3)	1.5 (1–3)	1.00
BMI	24.2 (19.4–30.4)	24.3 (19.5–33.9)	1.00
Type of accident			0.42
Bicycle	14 (73.7%)	14 (66.7%)	
Sports	2 (10.5%)	6 (28.6%)	
Fall of other cause	2 (10.5%)	1 (4.8%)	
Direct blow	1 (5.3%)	0 (0.0%)	
Fracture location			0.33
Lateral third	5 (26.3%)	3 (14.3%)	
Middle third	13 (68.4%)	18 (85.7%)	
Medial third	1 (5.3%)	0 (0.0%)	
Dislocation			1.00
<5 mm	1 (5.3%)	2 (9.5%)	
>5 mm	18 (94.7%)	19 (90.5%)	

**Table 2:** Distribution of block characteristics in the block group (*n* = 19).

Type of Block	
Supraclavicular Nerve block	17 (89.5%)
Intermediate cervical plexus block	2 (10.5%)
**Local anesthetic**	
Bupivacain 0.5% + Clonidin	17 (89.5%)
Ropivacain 1% + Clonidin	1 (5.3%)
Ropivacain 0.75%	1 (5.3%)
**Dose of local anesthetic**	
3 mL	5 (26.3%)
3.5 mL	1 (5.3%)
4 mL	12 (63.2%)
5 mL	1 (5.3%)

**Table 3:** The frequency and dosage of all pain medication given, stratified by intervention group. The frequency refers to the number of patients receiving the specific medication. The dose refers to the cumulative dose given over 24 h after admission. For the dose, mean and range are reported.

	Block	Control
Sample size, *n*	19	21
Metamizole—iv		
given	2 (10.5%)	5 (23.8%)
dose (mg)	1750 (500–3000)	2214 (1000–4000)
Ketorolac—iv		
given	0 (0.0%)	3 (14.3%)
dose (mg)	0 (0.0)	30 (30–30)
Paracetamol—iv		
given	3 (15.8%)	3 (14.3%)
dose (mg)	1333 (1000–2000)	2000 (1000–4000)
Ibuprofen—per os		
given	12 (63.2%)	18 (85.7%)
dose (mg)	633 (400–2000)	1156 (400–2200)
Metamizole—per os		
given	4 (21.1%)	15 (71.4%)
dose (mg)	2000 (1000–3000)	2607 (500–5000)
Paracetamol—per os		
given	1 (5.3%)	3 (14.3%)
dose (mg)	1000 (1000–1000)	1500 (1000–2000)
Oxycodone—per os		
given	7 (36.8%)	6 (28.6%)
dose (mg)	18 (7–35)	22 (7–40)
Fentanyl—iv		
given	4 (21.1%)	6 (28.6%)
dose (µg)	162 (50–300)	200 (50–350)

## Data Availability

Data are available from the authors upon reasonable request.

## Supplementary Materials

The following supporting information can be downloaded at: https://www.mdpi.com/article/10.3390/jcm14228249/s1, Figure S1: Typical double dot of the GAN (white arrows) after its loop around the lateral border of the SCM. The GAN is already located over the PVF. At this sonographic cross-section, the SCLN (dotted white circle) has not yet pierced the PVF completely and lies as a grape-like structure over the middle scalene muscle (mSM). The ventral ramus of the 5th spinal nerve (RvC5) lies ventrally of the mSM; Figure S2: After perforating the PVF, the SCLN divides into three bundles (three white arrows), while the PN (single white arrow) remains beneath the PVF, coursing around the anterior scalene muscle (aSM). C5 and C6 represent the ventral rami of their respective spinal nerves, in this patient separated by the scalenus minimus muscle (minSM). mSM: middle scalene muscle; SCM: sternocleidomastoid muscle; Figure S3: Injection of the local anesthetic (LA) using the in-plane technique around the supraclavicular nerves (SCLN, dotted white circle) strictly over the prevertebral fascia (PVF, upward white arrows). The injection needle (downward gray arrows) is advanced laterally between the posterior edge of the sternocleidomastoid muscle (SCM) and the PVF. At this point, the SCLN has already pierced the PVF, so the underlying ventral rami of the spinal nerves C5 and C6 are spared from the LA. Anterior scalene muscle: aSM, middle scalene muscle: mSM; Figure S4: The individual course of the pain intensity measurements over time. Post-op measurements are indicated by light colour. The order of patients corresponds to the date of study entry; Figure S5: The distribution of the time spent at a maximal pain level of 0, 1, 2, or 3 within the first 12 h stratified by the intervention group. The distributions are visualized by cumulative distribution functions; Table S1: The questions of the five patient experience measures and the response options. The 4th and 5th questions were only asked in patients from the block group.

## Abbreviations

The following abbreviations are used in this manuscript:

RA	Regional anesthesia
SCLN	Supraclavicular nerve
PN	Phrenic nerve
GAN	Great auricular nerve
TCN	Transverse cervical nerve
LON	Lesser occipital nerve
SCM	Sternocleidomastoid muscle
SCF	Superficial cervical fascia
PVF	Prevertebral fascia
mSM	Middle scalene muscle
aSM	anterior scalene muscle
minSM	Scalenus minimus muscle
RvC5	Ventral ramus of the 5th spinal nerve
CF	Clavicle fracture
ED	Emergency department
NRS	Numeric rating scale
ASA	American Society of Anesthesiologists
