# Supplementary material for: Ultrasound-Guided Supraclavicular Nerves Block for Acute Pain Management in Clavicular Fractures—A Pragmatic Randomized Trial
Source: J Clin Med. 2025 Nov 20;14(22):8249. doi: 10.3390/jcm14228249 (PMC12653940; doi:10.3390/jcm14228249)
Supplement: Supplementary file 1 [file jcm-14-08249-s001.zip › jcm-3962053-supplementary.pdf]

Ultrasound-guided Supraclavicular Nerves Block for acute Pain Management in Clavicular Fractures—A Pragmatic Randomized Trial

Eckehart Schöll, Mark Ulrich Gerbershagen, Werner Vach, Maria Rösli and Rainer Jürgen Litz

Supplementary Material

Supplementary Methods

S1. Detailed Sonoanatomy of the Supraclavicular Nerves

The supraclavicular nerves (SCLN) arise predominantly from the ventral ramus of C4, with minor contributions from C3 and C5. After originating from the superficial cervical plexus, the nerves course laterally and caudally before traversing the prevertebral fascia (PVF).

Key sonoanatomic landmarks include:

1. **Sternocleidomastoid muscle (SCM)** – forming the anterior border of the posterior cervical triangle.
2. **Prevertebral fascia (PVF)** – a hyperechoic linear structure covering the scalene muscles and separating the SCLN from the phrenic nerve.
3. **Anterior and middle scalene muscles** – deeper structures supporting identification of the PVF plane.
4. **SCLN cluster** – typically visible as small, hyperechoic fascicles immediately after they pierce the PVF.
5. **Surrounding sensory branches** – the great auricular nerve (GAN), transverse cervical nerve (TCN), and lesser occipital nerve (LON), which may lie within the same interfascial compartment after perforating the PVF.

The phrenic nerve (PN), in contrast, usually remains beneath the PVF, running along the anterior scalene muscle. This anatomical distinction is essential for selective blockade. See figures S1-3 below)

S2. Procedural Technique for Ultrasound-Guided SCLN Block (Step-by-step)

This procedural description reflects the technique applied in the trial.

1. Indication

Acute pain management in adult patients with clavicular fractures requiring initial treatment in the ED.

2. Contraindications

- Infection at puncture site
- Allergy to local anesthetics
- Coagulopathy
- Inability to cooperate
- Severe respiratory compromise (relative)

3. Equipment

- High-frequency linear transducer (10–18 MHz)
- Sterile ultrasound gel and probe cover
- 22G or 25G short-bevel needle (50–70 mm)
- Local anesthetic: bupivacaine 0.5%
- Optional adjuvant: clonidine
- Standard monitoring

4. Patient positioning

- Supine
- Head gently rotated to the contralateral side
- Ipsilateral arm resting comfortably

5. Probe placement

Place the transducer in the **posterior cervical triangle**, superficial to the SCM, with slight caudal tilt. Identify:

- The SCM
- The PVF
- The SCLN emerging as hyperechoic fascicles
- The anterior scalene muscle (landmark for PN)

6. Identification steps

1. Visualize the PVF as a distinct hyperechoic line.
2. Trace the SCLN cluster where it pierces the PVF.
3. Confirm separation of SCLN (extraprevertebral) and PN (subprevertebral).
4. Adjust depth and gain to optimize nerve visibility.

7. Needle approach

- In-plane, lateral-to-medial
- Always target the space superficial to the PVF
- Advance slowly, ensuring the needle tip remains above the PVF
- Aspirate frequently to avoid vascular injection

8. Injection

- Intended injectate: 3 ml bupivacaine 0.5% + 75 µg clonidine
- Confirm spread around the SCLN cluster without deep spread beneath the PVF
- Observe circumferential or "layer-like" distribution

- Adjust needle tip position if spread is insufficient

9. Troubleshooting

- SCLN not clearly identifiable:
  → consider intermediate cervical plexus block
- Reflux of injectate or deep spread:
  → withdraw slightly and redirect to remain above PVF
- Unexpected blockade of GAN/TCN:
  → likely due to interfascial spread; limit volume
- Loss of efficacy over time:
  → consider whether a small rescue dose is required

10. Expected onset

< 5 minutes, based on clinical experience and previous reports [23].

S3. Supplementary Figures

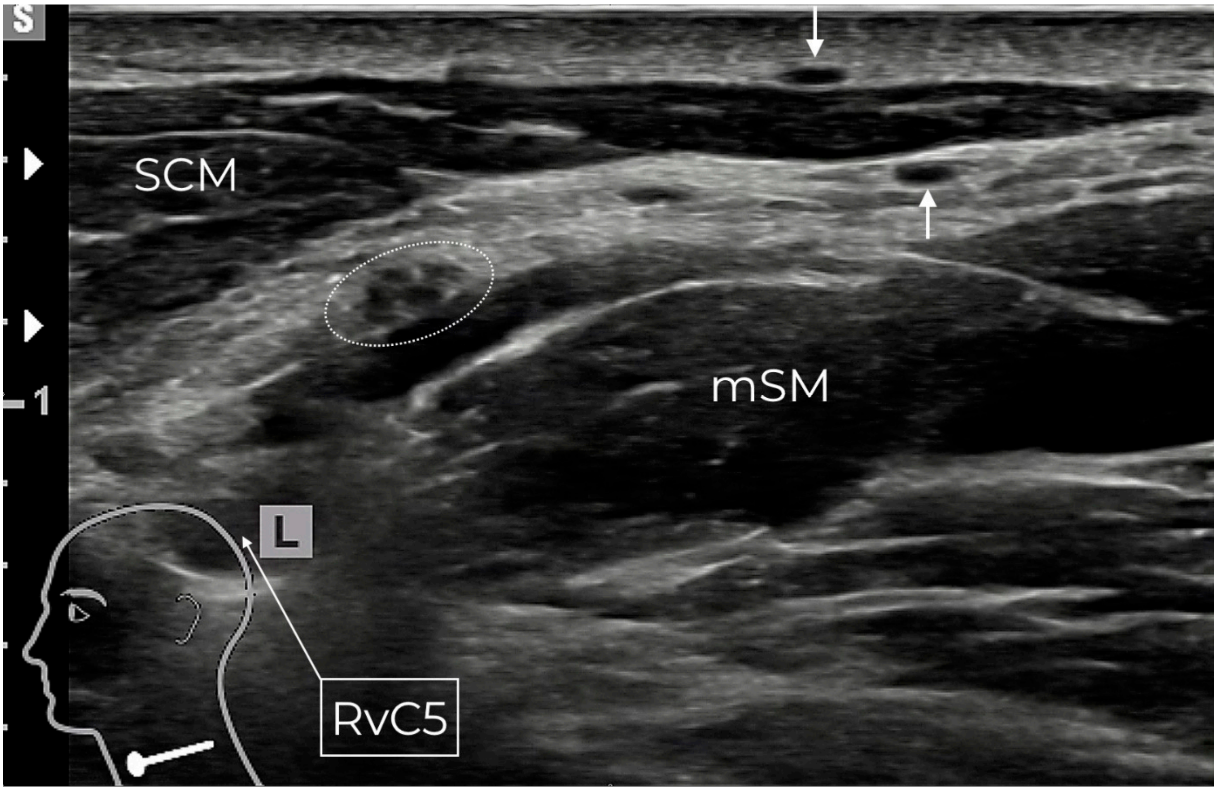

Figure S1: Typical double dot of the GAN (white arrows) after its loop around the lateral border of the SCM. The GAN is already located over the PVF. At this sonographic cross-section, the SCLN (dotted white circle) has not yet pierced the PVF completely and lies as a grape-like structure over the middle scalene muscle (mSM). The ventral ramus of the 5th spinal nerve (RvC5) lies ventrally of the mSM.

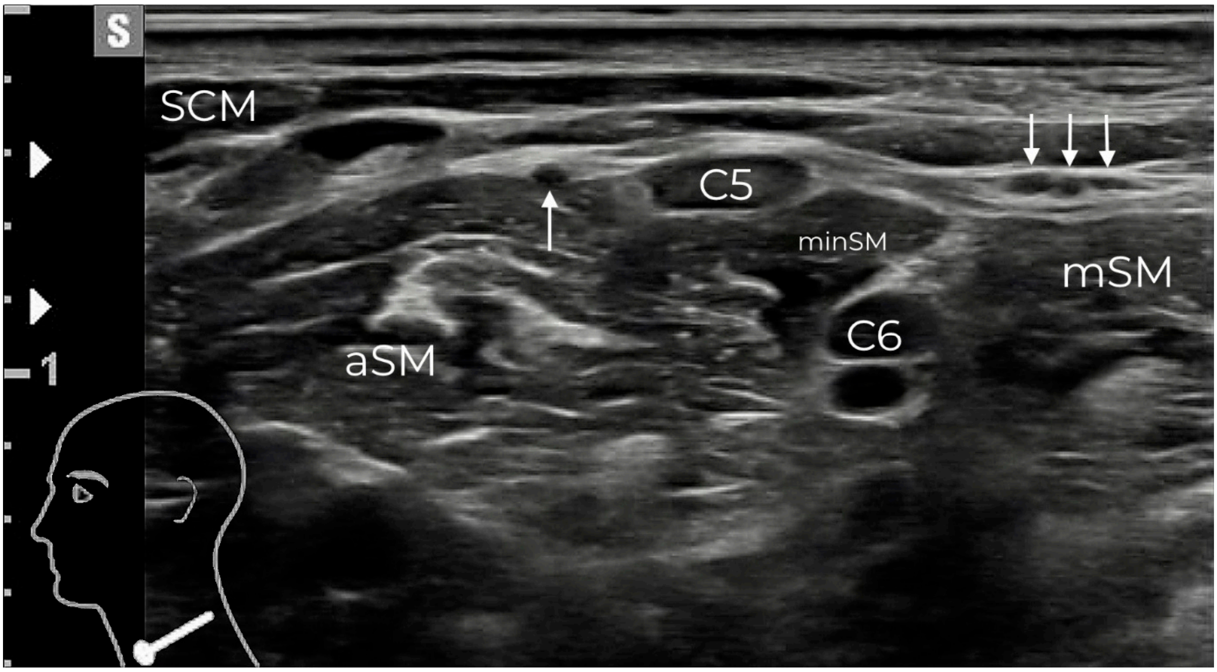

Figure S2: After perforating the PVF, the SCLN divides into three bundles (three white arrows), while the PN (single white arrow) remains beneath the PVF, coursing around the anterior scalene muscle (aSM). C5 and C6 represent the ventral rami of their respective spinal nerves, in this patient separated by the scalenus minimus muscle (minSM). mSM: middle scalene muscle; SCM: sternocleidomastoid muscle.

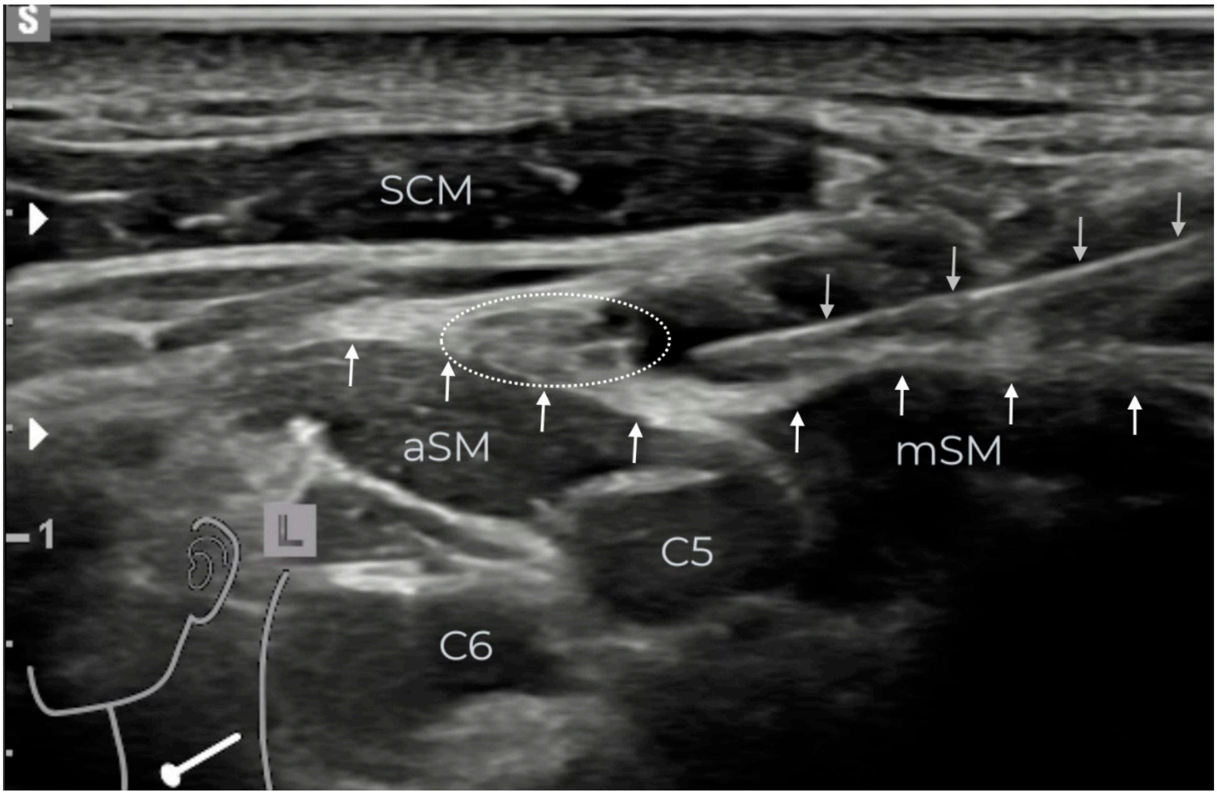

Figure S3: Injection of the local anesthetic (LA) using the in-plane technique around the supraclavicular nerves (SCLN, dotted white circle) strictly over the prevertebral fascia (PVF, upward white arrows). The injection needle (downward gray arrows) is advanced laterally between the posterior edge of the sternocleidomastoid muscle (SCM) and the PVF. At this point, the SCLN has already pierced the PVF, so the underlying ventral rami of the spinal nerves C5 and C6 are spared from the LA. Anterior scalene muscle: aSM, middle scalene muscle: mSM.

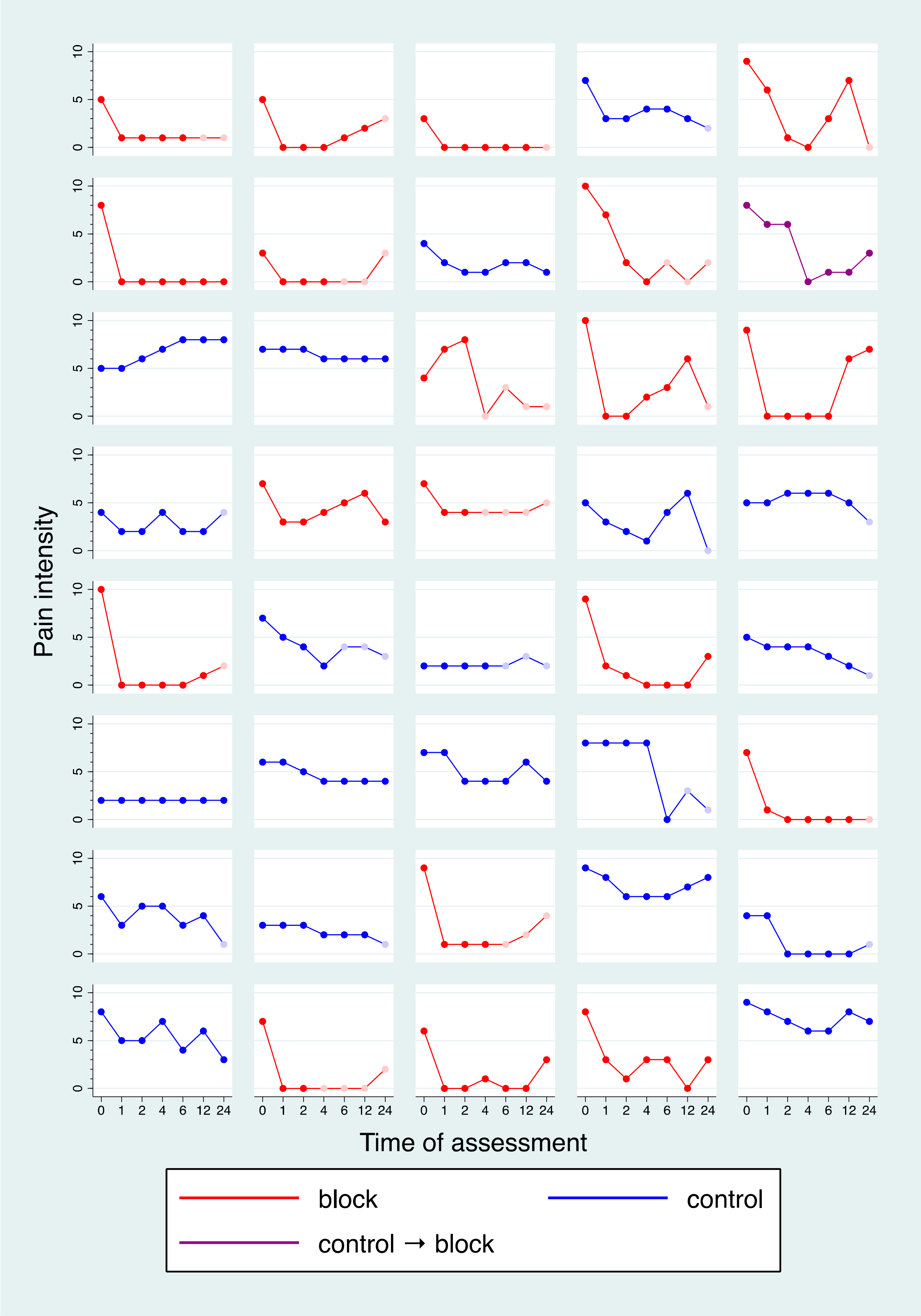

Figure S4: The individual course of the pain intensity measurements over time. Post-op measurements are indicated by light colour. The order of patients corresponds to the date of study entry.

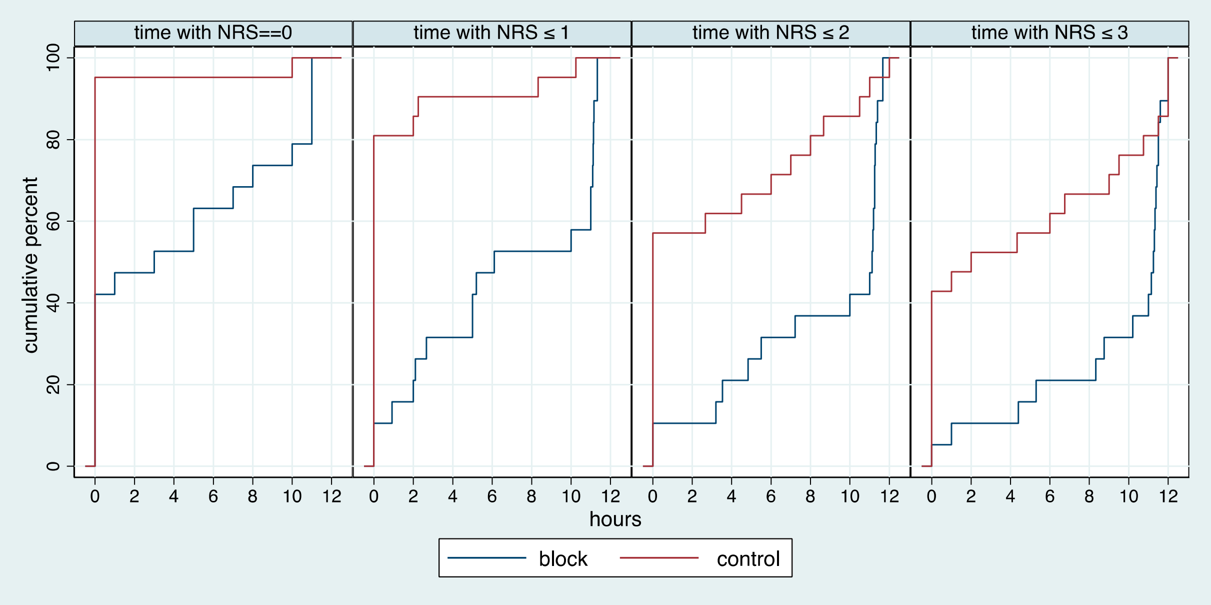

Figure S5: The distribution of the time spent at a maximal pain level of 0, 1, 2, or 3 within the first 12 hours stratified by the intervention group. The distributions are visualized by cumulative distribution functions.

Supplementary Table S1. Patient Satisfaction Questionnaire Used in the Trial

Question	Response options
Did you have any pain in the first 24 hours?	"no" "less" "moderate" "severe" "extreme"
Were you satisfied with the pain management on the ED?	"excellent" "good" "ok" "less satisfied" "dissatisfied"
Were you satisfied with the overall pain management at the clinic?	"excellent" "good" "fair" "less" "not at all"
How did you experience the procedure?	"excellent" "good" "ok" "unpleasant" "very unpleasant"
Would you have the block done again?	"yes" "probably" "don't know" "rather not" "no"

Supplementary Table S1: The questions of the five patient experience measures and the response options. The 4th and 5th questions were only asked in patients from the block group.
